# Insulin Matters: A Practical Approach to Basal Insulin Management in Type 2 Diabetes

**DOI:** 10.1007/s13300-018-0375-7

**Published:** 2018-02-23

**Authors:** Lori Berard, Noreen Antonishyn, Kathryn Arcudi, Sarah Blunden, Alice Cheng, Ronald Goldenberg, Stewart Harris, Shelley Jones, Upender Mehan, James Morrell, Robert Roscoe, Rick Siemens, Michael Vallis, Jean-François Yale

**Affiliations:** 1 Winnipeg Regional Health Authority, Winnipeg Diabetes Research Group, Health Sciences Centre, Winnipeg, MB Canada; 2 Department of Endocrinology, Alberta Health Services, Edmonton, AB Canada; 3 Diabetes Clinic, The Montreal West Island Integrated University Health and Social Services Centre (Lakeshore General Hospital), Pointe-Claire, QC Canada; 4 Diabetes Education, LMC Diabetes and Endocrinology, Montreal, QC Canada; 5 Division of Endocrinology and Metabolism, St. Michael’s Hospital, Toronto, ON Canada; 6 Trillium Health Partners, Credit Valley Hospital, Mississauga, ON Canada; 7 Department of Medicine, University of Toronto, Toronto, ON Canada; 8 LMC Diabetes and Endocrinology, Thornhill, ON Canada; 9 Department of Family Medicine, Western University, London, ON Canada; 10 Horizon Health Network, Moncton, NB Canada; 11 The Centre for Family Medicine, Kitchener, ON Canada; 12 Department of Family Medicine, McMaster University, Hamilton, ON Canada; 13 Diabetes Services, Island Health, Victoria, BC Canada; 14 Diabetes Education Centre, Saint John Regional Hospital, Saint John, NB Canada; 15 London Drugs Pharmacy, Lethbridge, AB Canada; 16 Behaviour Change Institute, Nova Scotia Health Authority, Halifax, NS Canada; 17 Department of Family Medicine, Dalhousie University, Halifax, NS Canada; 18 Department of Medicine, McGill University, Montreal, QC Canada

**Keywords:** Basal insulin, Glycemic target, Insulin initiation, Insulin titration, Patient barriers, Patient follow-up, Treatment delay, Type 2 diabetes

## Abstract

It is currently estimated that 11 million Canadians are living with diabetes or prediabetes. Although hyperglycemia is associated with serious complications, it is well established that improved glycemic control reduces the risk of microvascular complications and can also reduce cardiovascular (CV) complications over the long term. The UKPDS and ADVANCE landmark trials have resulted in diabetes guidelines recommending an A1C target of ≤ 7.0% for most patients or a target of ≤ 6.5% to further reduce the risk of nephropathy and retinopathy in those with type 2 diabetes (T2D), if it can be achieved safely. However, half of the people with T2D in Canada are not achieving these glycemic targets, despite advances in diabetes pharmacological management. There are many contributing factors to account for this poor outcome; however, one of the major factors is the delay in treatment advancement, particularly a resistance to insulin initiation and intensification. To simplify the process of initiating and titrating insulin in T2D patients, a group of Canadian experts reviewed the evidence and best clinical practices with the goal of providing guidance and practical recommendations to the diabetes healthcare community at large. This expert panel included general practitioners (GPs), nurses, nurse practitioners, endocrinologists, dieticians, pharmacists, and a psychologist. This article summarizes the panel recommendations.

## Basal Insulin Initiation

### Do We Still Need Insulin?

Type 2 diabetes (T2D) is a progressive disorder characterized by multiple pathophysiological defects. The core defects include insulin resistance in the muscle and liver and impaired insulin secretion due to β-cell failure [1, 2]. The progressive nature of the disease is such that it requires therapy to be intensified over time to compensate for the ongoing β-cell deficiency [2–4]. At the time of T2D diagnosis, more than 50% of β-cells have already been lost, and continue to decline at an average rate of 5% per year [1, 2, 5]. Therefore, the use of insulin is an appropriate option at any point in the management of T2D to *replace* the insulin that the pancreas is unable to produce sufficiently [1, 6]. In fact, when the maximum output of insulin has decreased to 15% or 20% of normal, non-insulin anti-hyperglycemic agents can no longer sustain glycemic control and insulin supplementation becomes a necessity [5]. The usual starting point for insulin therapy in T2D is with basal insulin owing to its simplicity and lower risk of hypoglycemia [7].

### When and in Whom to Initiate Insulin in T2D

The panel recommendations as to *when and in whom* to initiate insulin are summarized in Table 1.

**Table 1:** When and in whom to initiate insulin in T2D

When to consider insulin initiation	When NOT to initiate insulin
Maximally tolerated non-insulin agents but A1C above the individualized target (usually 7.0%) New diagnosis A1C ≥ 8.5% Metabolic decompensation End-organ failure Patients with previous or current gestational diabetes Acute illness Prolonged course of steroids Intolerance to oral medications Any time you consider this is an appropriate option for your patients from diagnosis onwards	There are no contraindications for the use of insulin but insulin may not be appropriate for: Some older, asymptomatic patients, who may not gain sufficient benefit because of short life expectancy People limited in their capacity (physical or cognitive) to manage their diabetes who are at greater risk of hypoglycemia

[8]; http://guidelines.diabetes.ca/fullguidelines; http://www.rcn.org.uk; https://www.rcn.org.uk/professional-development/publications/pub-002254

### What are the Barriers to Insulin Initiation?

Clinical inertia, defined as the failure on the part of the provider to advance therapy when required, adversely affects timely management of T2D [9–12]. Insulin is often initiated late in the course of the disease, after failure with multiple antihyperglycemic agents, and at glycemic values well above the recommended targets [11–15]. In Canada, mean A1C levels are > 8.5% and mean diabetes duration is ≥ 9 years before initiation of basal insulin in T2D patients [13, 15]. A UK retrospective study of pharmacologically treated T2D patients on one, two, or three oral antihyperglycemic agents reported that the median time to insulin initiation was > 7 years with an A1C ≥ 7.0% and the mean A1C levels at initiation was > 9.0% [12].

There are many barriers that contribute to this delay in initiation and intensification of insulin in T2D. It is important to emphasize that many of these barriers reflect the attitudes and beliefs of both patient and provider. Identifying and addressing both provider and patient beliefs and attitudes are therefore essential to mitigate those barriers (Tables 2 and 3).

**Table 2:** Provider barriers

Provider barriers	Panel recommendations to address provider barriers
Concerns about the risks to patients [3, 11, 16, 17]:	Recognize the low risk of hypoglycemia and weight gain with earlier use of basal insulin in T2D [18, 19]
Excess weight gain Hypoglycemia Impaired quality of life	Recognize the lower risk of hypoglycemia with each successive generation of basal insulin (human vs analogue vs next generation analogue) [14, 20–32]
Assumptions about patient inability to use insulin Assumptions about patient refusal to use insulin	Discuss with your patient. Do not assume that your patient is uninterested or non-adherent. Negotiate benefit versus risk or initiation versus inaction [3, 34]
	Awareness that most people can manage their treatment with appropriate education and support [3, 17]
[3, 11, 17, 33]	Diabetes education and allied support (cognitive behavior therapy and motivational communication) by healthcare professionals (HCPs) to improve adherence and health outcomes [16, 33–36]
Lack of resources [3, 11, 16, 17, 33]: Drug costs Availability of staff Skills needed to support insulin initiation Time	Include the family members/caregivers in the educational and ongoing support [34, 37, 38] Consider getting support from qualified team members or community [3, 34, 36] Utilize resources from Diabetes Canada including: The Insulin Prescription Tool: http://guidelines.diabetes.ca/bloodglucoselowering/insulinprescriptiontool and videos: http://guidelines.diabetes.ca/insulin
Reluctance to utilize insulin early in the diagnosis of T2D [9]	Recognize that delaying therapy prevents patients from achieving A1C targets and increases the occurrence of major diabetes complications [9, 12]

**Table 3:** Patient barriers [39–43]

Concern	Panel recommendations
Fear of needles or apprehension toward injections Feeling that insulin is too complicated [3, 11, 16, 33, 44]	Demonstrate injection technique: show the insulin pen and small needle tips. Apply the principles of systematic desensitization (self-controlled exposure) Highlight that the injection is into subcutaneous tissue, not a vein Invite patient to try these without insulin, in your office (i.e., dry injection); give first injection together with patient to observe, support and ensure correct administration of insulin *Educating on injections*: see http://www.fit4diabetes.com/canada-english/fit-recommendations/
Feeling that this is a personal failure [3, 11, 16, 33, 45]	Pro-action. Do not wait to talk about insulin once the patient needs insulin. Explain from the time of diagnosis that insulin is a likely treatment option in the course of T2D [46] Discuss with the patient, using decisional balance analysis (pros and cons), that need to advance therapy is due to the progressive nature of diabetes, not because the patient has done something wrong
Belief that insulin causes diabetes complications [3, 11, 16, 33]	Insulin is a natural hormone and a replacement therapy [42] Explain why insulin becomes necessary for most patients with diabetes eventually; it is not a punishment [46] Explain that use of insulin will help achieve glycemic target and minimize the risk of complications [47, 48]
Concerns over hypoglycemia (BG < 4.0 mmol/L) [3, 11, 16, 33, 49–52]	Reassure the patient that most hypoglycemic episodes are mild.* Severe* hypoglycemia (defined as requiring assistance by another person) is relatively rare^a^ [52] http://guidelines.diabetes.ca/browse/chapter14; [53] http://guidelines.diabetes.ca/browse/chapter13; [20, 29, 42, 47, 54] Educate the patient on how to recognize and respond to symptoms [52] http://guidelines.diabetes.ca/browse/chapter14 Make sure the patient and partner/family (if applicable) know how to recognize, treat, and avoid hypoglycemia, and how to self-adjust insulin [34] Choose insulins and regimens with lower rates of hypoglycemia [14, 29] Use systematic desensitization to allow the patient to work from a psychologically safe zone to a medically safe zone
Concerns over weight gain [3, 11, 16, 33]	Encourage healthy diet and moderate exercise. Monitor weight. http://guidelines.diabetes.ca/fullguidelines Combine insulin with metformin or other NIAHA with weight benefit. http://guidelines.diabetes.ca/cdacpg_resources/CPG_Quick_Reference_Guide_WEB.pdf [14, 29] Explain that weight gain with basal insulin regimens is small especially with newer basal insulin analogues (1–2 kg) [14, 20, 26–29, 32]
Belief that insulin can never be stopped and will restrict lifestyle [3, 4, 16, 33, 42]	Offer a 3-month trial period with subsequent reassessment. http://guidelines.diabetes.ca/fullguidelines Recall that engaging the patient in the decision empowers them and leads to better outcomes [3, 4] Prescribe once-daily basal insulin that minimizes inconvenience and is easy to use. http://guidelines.diabetes.ca/bloodglucoselowering/insulinprescriptiontool

*NIAHA* non-insulin anti-hyperglycemic agent, *BG* blood glucose

^a^In UKPDS, the annual incidence of severe hypoglycemia in insulin-treated patients was < 3%. With the newer long-acting basal insulins this is even lower (2.3%) [47]

### What is Your Role in Insulin Therapy?

Success in overcoming patient barriers relies greatly on listening to the patient and proactively addressing their fears and concerns [55, 56]. Open dialogue with the patient throughout the continuum of diabetes management, with an emphasis on the positive benefits of insulin therapy, will significantly enhance the outcomes for patients with diabetes. See Table 4 for review of action points with your patient.

**Table 4:** A new *LEASE* on insulin management [55]

Listen and ask	Actively listen to fears and concerns. Normalize these concerns before discussing alternatives Invite discussion, show conviction of belief and supportive body language
Educate	Ask permission to educate about the importance of insulin, the progressive nature of the disease, how to self-manage their disease
Address	Proactively address patient concerns that may deter initiation and adherence to insulin Ask questions, identify the barriers, outline goals
Support	Enlist support of diabetes management team Provide continuous support and education through the course of treatment
Empower	Encourage and educate the patient on self-management: demonstrate how the pen works and let them try it, explain how to take medications, how to self-monitor blood glucose, how to prevent and treat hypoglycemia, reinforce healthy lifestyle and diet Be comfortable with the principle of shaping: in other words, with repetition and support for next step goals, self-efficacy in a new behavior can develop

## Basal Insulin Dose and Titration Recommendations

In light of the persistent barriers contributing to delays in diabetes management with insulin, there is an urgent need for a simplified and practical approach to the initiation and intensification of insulin. Complex regimens and unrealistic targets can worsen the patient’s engagement in the process and ultimately the patient’s well-being [3, 4, 57]. Simplification allows for empowerment by engaging the patient in doable tasks, which provides the context for behavior shaping (next step goals) and self-efficacy (confidence in the face of barriers) [58].

### What Do We Want in a Basal Insulin Recommendation?

- A starting dose that can be safely applied and individualized.
- A titration schedule that is simple and can be safely patient-driven, with a fasting blood glucose (FBG) target that can be individualized. Patient-driven titration schedules are as effective as provider-driven titration schedules [19, 59–64] and engage the patient, which in turn can lower barriers to insulin therapy [4, 65, 66].
- Clear instructions to the patient on how the dose will be titrated, to manage expectations which will empower the patient and improve adherence to therapy [3, 4, 16, 66].
- Recognition that insulin initiation and titration are two separate behaviors for the patient, each of which needs to be addressed in relation to patient readiness to change.

### How to Select a Basal Insulin?

Three generations of basal insulins are available in Canada. The first generation of basal insulin is NPH, a human insulin that has been available for many decades, since 1946. The basal analogues (insulins detemir and glargine (Gla-100)) emerged in the 2000s and provided longer duration of action, improved day-to-day variability, reduced hypoglycemia, especially nocturnal, and did not require resuspension (as does NPH) [67]. A next generation of long-acting basal insulins—insulins glargine 300 U/mL (Gla-300) and degludec—have emerged with an extended action profile, improved safety, and the advantage of being administered in smaller volumes [29]. Table 5 summarizes the main characteristics of the currently available basal insulins. The panel recognizes that the choice of basal insulin may depend on access, cost, and clinical judgment with respect to the patient’s individual needs and lifestyle [29].

**Table 5:** Basal insulins

Insulin classification		Duration of action	CV safety	Risk of nocturnal hypoglycemia	Considerations
Intermediate-acting	NPH	~ 18 h	–	+++	Needs resuspension Administered usually twice daily
Long-acting	Detemir	16–24 h	–	++	Administered once or twice daily
	Gla-100	~ 24 h	Demonstrated (neutral)	++	Administered once daily, same time of day Available in a fixed-ratio combination with lixisenatide
Next generation	Gla-300 (U300)	~ 30 h^b^	Demonstrated^a^ (neutral)	+	Smaller volume (U300) Administered once daily Flexible +
	Degludec (U100, U200)	~ 30 h^b^	Demonstrated (neutral)	+	Option smaller volume (U200) Administered once daily Flexible ++ U100 available in a fixed-ratio combination with liraglutide

Duration of action and considerations: http://guidelines.diabetes.ca/fullguidelines/chapter12; [14, 29, 68–78]. Degludec and Gla-300 studies: [18, 20–28, 30–32, 70, 79, 80]

*Gla-100* glargine 100 U/mL, *Gla-300* glargine 300 U/mL

+ Insulins with low risk of hypoglycemia; ++ Insulins with moderate risk of hypoglycemia; +++ Insulins with higher risk of causing hypoglycemia

^a^Based on results from ORIGIN with Gla-100

^b^PK/PD studies at 0.4 U/kg

### How to Dose?

There are several important concepts to remember when dosing basal insulin: (a) the starting dose will be wrong; (b) there is no maximal insulin dose; (c) titration of insulin dose is the key [8]. Each of these concepts needs to be explicitly discussed and understood by the patient in order for titration to be successful. Despite 92% of physicians agreeing that “insulin intensification is an essential element of diabetes management,” 30% of primary care physicians “never or rarely” personally intensified insulin (vs 4% of specialists) in the multinational survey MODIFY [14, 81]. Interestingly, in a recent multinational survey, HCPs generally preferred a gradual and safe approach to titration to avoid hypoglycemia whereas patients are frustrated by time to reach goal [66]. It is therefore important to manage the patient’s expectations.

The starting dose for basal insulin recommended by this panel is 10 U/day. The dose should be incrementally increased on a regular basis using target FBG as the determinant for dose adjustments. At initiation, educating patients that many people will need at least 40–50 units of basal insulin to achieve target FBG is useful for goal setting and behavior shaping. This may help mitigate patient fear/reluctance to up-titrate [8].

Box 1A details the recommendations by the panel for basal insulin dose and titration.

Box 1B provides a summary of key recommendations, including a starting dose and titration schedule.

### Basal Insulin Dose and Titration Recommendations (2017)

**Table:** Box 1A: 2017 recommendations by the panel for basal insulin dose and titration

	Panel recommendations	Comments
The initial dose^a^	10 U/day [19, 22, 59] http://guidelines.diabetes.ca/browse/appendices/appendix3 Other considerations: 0.2 U/kg/day [68, 82] Using FBG as starting point: e.g., if FBG is 16 mmol/L start at 16 U [59]	May need to be lower for some patients—recall that the starting dose should be individualized [14] http://guidelines.diabetes.ca/cdacpg_resources/CPG_Quick_Reference_Guide_WEB.pdf The lower dosages have the advantage of decreasing the risk of a hypoglycemic reaction with the first injection, but make the titration period a bit longer Discuss and negotiate your patient’s expectation
Fasting SMBG target	Target should be 4.0–7.0 mmol/L for most people Patient/HCP contact recommended at 7.0 mmol/L. HCP may then suggest continuing to 4.0–5.5 mmol/L [19, 20, 59, 80, 83–85]	Individualize target with a step approach (within 3 months) [14] http://guidelines.diabetes.ca/cdacpg_resources/CPG_Quick_Reference_Guide_WEB.pdf Important to educate that diabetes is a progressive disease and this is a moving target [4]
Dose adjustments	Select a simple titration algorithm that matches patient lifestyle [57] The following dose adjustment algorithms have been shown to be safe and effective. Select the one that is easiest for the patient to follow: One easy titration algorithm is 1 unit every day^b^ [19, 63, 64, 66] Other titration algorithms include: 2 units twice weekly based on lowest fasting SMGB value of the last 3 days [26, 27, 62, 86] Every week, based on lowest fasting SMGB value of the last 3 days [26, 63, 64] *Other considerations*: If (nocturnal) hypoglycemia occurs (BG < 4.0 mmol/L) reduce the dose by 2–4 units, or 10% of the basal dose based on clinical judgement [57] For other considerations, see Table 6	Measure glucose level at least every morning before breakfast^c^ [57] http://guidelines.diabetes.ca/browse/appendices/appendix3 Remind patient to adjust the basal insulin based on morning glucose not bedtime glucose^c^ [57] Assess for possible hypoglycemia (< 4.0 mmol/L) and decrease titration [52] http://guidelines.diabetes.ca/fullguidelines/chapter14 Recognize that patient fear of hypoglycemia is easily elicited (hypoglycemia is a traumatic stress) and that providers underestimate the psychological impact of nonsevere hypoglycemia [51] *Mitigating hypoglycemia*: Is there an identifiable cause? [52] http://guidelines.diabetes.ca/fullguidelines/chapter14 Teach patients how to prevent, recognize, and treat hypoglycemia [52] http://guidelines.diabetes.ca/fullguidelines/chapter14 Confirm with patient that it is not “*pseudo*-*hypoglycemia*”. Explain what pseudo-hypoglycemia^d^ is and ways to mitigate it [54] If no identifiable and preventable cause is identified, reduce the dose Confirm patient is using an accurate glucometer
Optimal/maximum basal insulin dose	Educate the patient of their expected dose [3, 57] In most studies: 40 to 50 units is needed [8, 19, 26, 27, 66] Communicate how long it will take them to reach target (e.g., if the expected dose is 60 units at 1 U/day increase, then it will take on average 6 weeks)	Indication that basal insulin is not enough includes: Up-titrations without a corresponding drop on BG (verify patient adherence and check injection sites). http://www.fit4diabetes.com/canada-english/fit-recommendations/ Patient has surpassed 1 U/kg/day of basal insulin without sufficient FBG control [87] FBG in target, but A1C above target

*BG* blood glucose,* FBG* fasting blood glucose,* SMBG* self-monitored blood glucose

^a^For more information on how to handle any oral agents and other FAQs, see Tables 6 and 7

^b^Algorithm proven safe and effective with insulin glargine 100 units/mL (Lantus^®^) and 300 units/mL (Toujeo™)

^c^Adjust accordingly if shift worker

^d^Pseudo-hypoglycemia: an event in which the patient experiences symptoms of hypoglycemia with a BG > 3.9 mmol/L but approaching that level [54]

**Figure:**
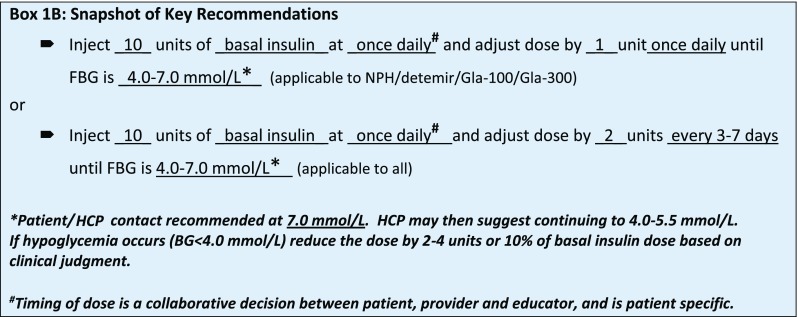

### Frequent Questions and What to Do with Previous Drugs When Initiating Basal Insulin

Tables 6 and 7 outline some of the frequently encountered questions and concerns facing HCPs when initiating and titrating basal insulin.

**Table 6:** Frequently asked questions and concerns

Question	Answer
Is 4.0 to 7.0 mmol/L too aggressive?	Depends on individual target and patient characteristics (e.g., younger patient, patient with established retinopathy/nephropathy, etc.). http://guidelines.diabetes.ca/cdacpg_resources/CPG_Quick_Reference_Guide_WEB.pdf
Is there a ceiling to titration process?	There is no such thing as a maximum dose [8] Consider resuming titration when FBG values are above patient-agreed target for 3 consecutive days; resume 1 unit daily titration unti FBG < 7.0 mmol/L is reached without hypoglycemia Patient/HCP contact recommended at 7.0 mmol/L
What to do if daytime hypoglycemia occurs while on secretagogues?	Dose adjustment of secretatogue and/or basal insulin recommended If on NPH, consider basal analogue
When is it appropriate to intensify treatment with another agent?	When A1C level remains above individual target after 3–6 months despite appropriate treatment initiation and optimization have occurred or insulin dose is > 1.0 U/kg/day. http://guidelines.diabetes.ca/cdacpg_resources/CPG_Quick_Reference_Guide_WEB.pdf
What to do if sickness occurs?	Normally continue with the usual dose of basal insulin Test more frequently If problems eating or hydrating: stop metformin, SGLT2 inhibitor, insulin secretagogue, ACE inhibitor, ARBs, diuretic, NSAIDs Use SADMANS http://guidelines.diabetes.ca/browse/appendices/appendix7_2015. Complete the card (accessed by clicking on the link) and give it to your patient, including when to call and whom to reach for support [88]
What to do if patient has recently been hospitalized for a few days?	Verify if the dosages were modified during the hospitalization. The dosages are often decreased as the patient eats hospital food, and must often be increased back towards the previous dosages
What to do if unsure whether the dose was given?	Do not give the dose if unsure Test more frequently If values rise, may consider giving half the dose [88] *Additional comments*: Suggest using supportive tools or an insulin pen that has a memory feature that will indicate if the dose was given and when
What to do if gave the dose twice?	Test more frequently Take extra snack at bedtime Wake up every 2–3 h to test glucose. If < 7.0 mmol/L, take an extra snack [88] *Additional comments*: Check available resources in area: For example, call a nurse for advice, diabetes educator available for support, a 24 h pharmacy for a pharmacist’s advice Phone an “on-call” service and consider referral to ER
What to do if missed a dose?	If < 6 h: take usual dose (be aware of potential increase in risk of hypoglycemia with next injection) If 6–12 h: take 50% of normal dose If > 12 h: consider omitting dose or give 50% when remember and 50% next dose and resume as per usual dosing administration schedule [89] *Additional comments* Recall that new long-acting basal insulins provide greater flexibility [24, 86]
Does insulin stacking (build-up of insulin in the circulation) occur with the long-acting basal insulins? [90]	No, there will be a steady state reached. The steady state will take longer to reach the longer the half-life of the insulin, minimizing the fluctuations in insulin levels [90]
When to consider seeking support from other HCPs? [87]	Patient has surpassed 1 U/kg/day of basal insulin without sufficient FBG control Patient has recurrent episodes of hypoglycemia Patient lacks engagement in the titration process. It is important to explore reasons for lack of engagement by screening for diabetes distress
When to refer to a specialist? [87]	Patient has frequent episodes of unexplained hypoglycemia Patient experiences complications (allergic reactions, lack of treatment response, edema, etc.) A1C level remains above individual target after 3–6 months despite appropriate treatment initiation and optimization have occurred At any point when comfort level is exceeded with available resources. It should be openly acknowledged that if either the patient or provider thinks they are “in over their head,” accessing additional resources is appropriate

*SGLT2* sodium-glucose co-transporter 2, *ACE* angiotensin-converting enzyme, *ARB* angiotensin receptor blockers, *NSAIDs* non-steroidal anti-inflammatory drugs

**Table 7:** What to do with previous drugs [8, 57, 91, 92]: usually continue all current anti-hyperglycemic agents when initiating basal insulin

Anti-hyperglycemic agent	Anti-hyperglycemic agents when initiating basal insulin	Comments	
Metformin	Continued	–	
Insulin secretagogues (meglitinide and sulfonylurea (SU))	Options to continue, reduce, or stop the sulfonylurea [7, 8, 93] Option to continue, reduce, or stop meglitinide [8]	If SU is stopped or reduced, titration of insulin is even more important When stopping SUs: Patients may need more insulin or go beyond basal insulin as glucose levels may go higher As a guideline, stopping SU is equivalent to about 20 U of insulin. Individual results necessitate monitoring and titration [94]	
TZDs	Usually discontinued^a^ [95]	Due to increased risk of edema and heart failure with insulin [8, 96]	
Incretin agents (GLP-1R agonist, DPP4i)	Continued^a^ [8, 97]	–	
SGLT2 inhibitor	Continued	–	

*GLP-1R* glucagon-like peptide-1 receptor, *DPP4i* protease dipeptidyl peptidase-4 inhibitor, *SGLT2* sodium-glucose co-transporter 2, *TZD* thiazolidinedione

^a^Recommendation to decrease TZDs is not indicated in Canada; linagliptin use with insulin is off-label (Trajenta^®^)

## Patient Support and Medical Follow-up

### How to Ensure Success of Basal Insulin Management?

The success of basal insulin initiation and titration relies not only on identifying and addressing the patient and practitioner barriers but also on contact frequency with the patient. Post-initiation follow-up may occur by many means including via phone, text, email (depending on jurisdiction), cloud, or virtual consult. Regular contact presents an opportunity to provide or revisit diabetes education, to provide support to patients on how to effectively self-manage their disease and to identify any causes of concern [3, 34]. Furthermore, titration should be revisited when the patient is not achieving goal, hypoglycemia occurs, or there is a change in the insulin type or brand (e.g., biosimilar) [87].

The panel provides guidelines for medical follow-up with patients in Box 2.

### Panel Recommendations for Medical Follow-up with Diabetes HCPs

**Table:** Box 2: panel recommendations for medical follow-up with diabetes HCPs [87, 91]

When	What and why
24–72 h	When initiating insulin or titration Support insulin initiation and reinforce titration
1–2 week(s)	Patients report BG readings Ensure titration is occurring normally
1 month	Patients report BG readings Ensure titration is occurring normally (it is encouraged to continue with biweekly contacts thereafter)
3 months	A1C measurement If not at goal, patient may continue with titration for another 3 months This contact point should occur in person or by virtual consult
6 months	A1C measurement Follow-up of titration If A1C above target, review glycemic profile and consider adding mealtime insulin
Within 24 h of hypoglycemia	Educate patient on recognizing, preventing, and treating hypoglycemia If recurrent hypoglycemia occurs, re-evaluate titration schedule or reduce dose (frequent, recurrent hypoglycemia is typically defined as 1–2 lows in 1 week)

## Conclusion

Several factors underlie the importance of the initiative put forth by this expert panel: there is a rising prevalence of diabetes [98]; half of the T2D population is not at target, among which 61% were receiving insulin therapy [99], suggesting delayed insulin initiation and intensification; there are multiple titration algorithms to choose from which adds to the confusion and complexity for patients and providers; and the arrival of new long-acting basal insulins and other pharmacological and technological advances that require consideration. This document was developed by a multidisciplinary panel to address frequently asked questions on insulin initiation and titration, and it establishes simple and practical guidelines for diabetes HCPs for effective initiation and titration of basal insulin, with the intent that it may translate to effective glycemic outcomes in clinical practice.

### Compliance with Ethical Guidelines

This article is based on previously conducted studies and does not contain any studies with human participants or animals performed by any of the authors.
